# Assessment the Bond Strength of Ceramic Brackets to CAD/CAM Nanoceramic Composite and Interpenetrating Network Composite after Different Surface Treatments

**DOI:** 10.1155/2018/1871598

**Published:** 2018-05-30

**Authors:** Mustafa Mehmet Özarslan, Özlem Üstün, Ulviye Sebnem Buyukkaplan, Çağatay Barutcigil, Nurullah Türker, Kubilay Barutcigil

**Affiliations:** ^1^Department of Prosthodontics, Akdeniz University Faculty of Dentistry, 07058, Antalya, Turkey; ^2^Department of Restorative Dentistry, Akdeniz University Faculty of Dentistry, 07058, Antalya, Turkey

## Abstract

Adult orthodontics may confront problems related to the bonding performance of orthodontic brackets to new generation restorative materials used for crown or laminate restorations. The aim of the present study was to investigate the shear bond strength of ceramic brackets to two new generation CAD/CAM interpenetrating network composite and nanoceramic composite after different surface treatments. Er,Cr:YSGG Laser, hydrofluoric acid (9%), sandblasting (50 *μ*m Al_2_O_3_), and silane were applied to the surfaces of 120 CAD/CAM specimens with 2 mm thickness and then ceramic brackets were bonded to the treated surfaces of the specimens. Bond strength was evaluated using the shear bond strength test. According to the results, CAD/CAM block types and surface treatment methods have significant effects on shear bond strength. The lowest bond strength values were found in the specimens treated with silane (3.35 ± 2.09 MPa) and highest values were found in the specimens treated with sandblast (8.92 ± 2.77 MPa). Sandblasting and hydrofluoric acid surface treatment led to the most durable bonds for the two types of CAD/CAM blocks in the present study. In conclusion, different surface treatments affect the shear bond strength of ceramic brackets to CAD/CAM interpenetrating network composite and nanoceramic composite. Among the evaluated treatments, sandblasting and hydrofluoric acid application resulted in sufficient bonding strength to ceramic brackets for both of the CAD/CAM materials.

## 1. Introduction

An important part of facial esthetics is dental esthetics, including adult orthodontics [1, 2]. The advanced age of an adult orthodontic patient may necessitate some partial and full coverage dental restorations such as jacket crowns or laminates alongside orthodontic treatment. Adult orthodontics may confront problems related to the bonding performance of orthodontic brackets to different restorative materials used for crown or laminate restorations such as ceramics or new generation CAD/CAM materials [3–6].

Dental ceramics remain the best dental material in terms of their biocompatibility and esthetic properties in contemporary fixed prosthodontics. However, dental ceramics have some disadvantages related to their physical properties such as brittleness, antagonistic tooth wear, and also bracket bonding problems. These disadvantages have led dental material scientists to develop materials that mimic the natural tooth structure and eliminate the disadvantages of conventional dental materials. Besides zirconia and conventional dental ceramics, various new generation dental materials such as interpenetrating network composite (IPN) and nanoceramic composite (NCC) are now also available to dental clinicians.

In the dental literature, different surface treatment methods were suggested to strengthen the bonding between brackets and dental restorative materials especially dental ceramics. Saraç et al. [4] evaluated the effectiveness of air-particle abrasion and tribochemical silica coating on the bond strength of feldspathic, leucite reinforced, and fluorapatite dental ceramics to brackets. They concluded that chair side tribochemical silica coating increased bond strength values in all groups. Besides this, leucite reinforced dental ceramic displayed better bond strength values than the other two types of dental ceramics used in the study [4]. In a study [5], bond strengths of a feldspathic dental ceramic to brackets were evaluated using hydrofluoric acid, silane, alumina sandblasting, and silica-coating applications. According to the results of the study [5], all the methods fulfilled the threshold of ideal bond strength for clinical use except the hydrofluoric acid alone. In the same study, the tribochemical silica-coating technique showed the highest bond strength; however, failure modes resulted in fractures of ceramic surfaces.

Since both IPN and NCC are relatively new in the dental market, studies related to their performance in different areas such as bonding features are ongoing [7]. For an adult patient having partial and full coverage crown restorations and needing orthodontic treatment, the bonding performance of ceramic brackets to these restorations remains unclear. Thus, the aim of the present study was to investigate the shear bond strength (SBS) of ceramic brackets to both IPN and NCC after different surface treatments including laser, sandblasting, and hydrofluoric (HF) acid conditioning.

## 2. Materials and Methods

For the present study, the SBS of an IPN [Vita Enamic (VE); Vita Zahnfabrik, Cuxhaven, Germany] and an NCC [Lava Ultimate (LU); 3M ESPE, St. Paul, MN, USA] to mandibular incisor ceramic brackets (Damon Clear; Ormco, Orange, CA, USA) were investigated. For this study, 120 specimens (60 VE and 60 LU) with the dimensions of 7 × 6 × 2 mm were sliced using a water cooling diamond blade with a low-speed cutting saw (Isomet 1000; Buehler, Lake Bluff, IL, USA) from VE and LU blocks. All specimen surfaces were finished and polished using the kits and instruments suggested by the manufacturers. The superficies of the specimens were abraded with carborundum sandpaper. For polishing of VE specimens, instruments of Technical Kit for VE (Vita Zahnfabrik, Cuxhaven, Germany) were applied with constant and stroking motions in a single direction under dry conditions. First, silicon carbide pink rubber disc was used with rotational handpiece at the speed of 10.000 rpm (NSK, Tokyo, Japan). After the use of pink rubber disc, grey disc with lesser particle size of the Technical Kit was used with 5000 rpm (NSK, Tokyo, Japan) as suggested by the manufacturer. For polishing of LU specimens, first step was performed with rubber of the luster set for LU (Meisinger Polishing Set, 3M ESPE, St. Paul, MN, USA) with 10.000 rpm (NSK, Tokyo, Japan). After that, using a soft bristle brush, polishing agent was applied to the specimen surfaces and polished using a soft polishing brush with 10.000 rpm (NSK, Tokyo, Japan). Finally, a cotton buff was used for complete surface polishing for LU with 10.000 rpm (NSK, Tokyo, Japan). The polishing procedures were continued for each of the specimens until the measurement of at least 0.2 *μ*m surface roughness value (*Ra*) which was the mean of 3 measurements from each specimen surface determined by a tactile profilometer (Surftest SJ 201, Mitutoyo, Tokyo, Japan). Then, each of the specimens was embedded in a rectangular acrylic resin (Paladent; Heraeus Kulzer, Grüner Weg, Hanau, Germany) with using silicone mold prepared specifically for the present study leaving one surface of the specimen exposed for surface treatments and bonding of the ceramic bracket. All specimen surfaces to which the ceramic brackets were to be bonded were outlined with a waterproof marker to identify the area to be treated. Materials used in the present study were given in Table 1.

### 2.1. Surface Treatments

Ten subgroups (*n* = 12) according to the surface treatments applied onto the specimens were formed by subdividing the 120 specimens from VE (*n* = 60) and LU (*n* = 60) as follows.

1 W Laser Group (1L): 2780 *η*m wavelength with a pulse duration of 140 to 200 *μ*s and a repetition rate of 10 Hz radiation was applied on the specimens by an Er,Cr:YSGG laser (Waterlase iPlus; Biolase Technology Inc., Irvine, CA). The energy density of the laser was 71 J/cm^2^. A 600-*μ*m diameter laser optical fiber was adjusted to be perpendicular to the CAD/CAM specimen surface, which scanned over the sample area for 20 seconds at a 1 mm distance. During the irradiation, the energy parameters applied continuously were 1 W, with airflow of 55% and water flow of 65%. After laser application, all specimens were rinsed with distilled water and were then air-dried.

2 W Laser Group (2L): 2780 *η*m wavelength with a pulse duration of 140 to 200 *μ*s and a repetition rate of 10 Hz radiation was applied on the specimens by an Er,Cr:YSGG laser (Waterlase iPlus; Biolase Technology Inc., Irvine, CA). The energy density of the laser was 71 J/cm^2^. A 600-*μ*m diameter laser optical fiber was adjusted to be perpendicular to the CAD/CAM specimen surface, which scanned over the sample area for 20 seconds at a 1 mm distance. During the irradiation, the energy parameters applied continuously were 2 W, with airflow of 55% and water flow of 65%. After laser application, all specimens were rinsed with distilled water and were then air-dried.

Al_2_O_3_ Sandblasted Group (SB): specimen surfaces were sandblasted (Blastmate II; Ney, Yucaipa, CA, USA) with 50 *μ*m Al_2_O_3_ for 20 s; 2-bar pressure was maintained for air abrasion. Specimens were mounted in a special holder forming right angles where the distance between the nozzle and the surface was 10 mm. The specimens were cleaned in distilled water and were then air-dried.

Silane Group (S): silanization (Ultradent Silane; Ultradent Product Inc., South Jordan, UT, USA) was performed for 60 seconds.

HF Acid Group (HF): the finished specimen surfaces were etched with 9% HF acid (Ultradent Porcelain Etch; Ultradent Product Inc., South Jordan, UT, USA) for 1 min. HF acid was rinsed off with a copious amount of water, and the surface was air-dried.

### 2.2. Bonding Procedure

Ceramic standard edgewise mandibular incisor brackets (Damon Clear; Ormco, Orange, CA, USA) were used in the present study. The ceramic brackets were bonded to the surface of specimens using a light-cured orthodontic adhesive (Transbond XT 3M; Unitek, Monrovia, CA, USA) according to the manufacturer's recommendations. Before bonding surface was light-cured, the excessive resin cement was removed. Light curing was performed using a light-emitting diode (VALO LED; Ultradent Product Inc., South Jordan, UT, USA) for producing high-intensity light at 395–480 nm, at the power of 1000 mW/cm^2^ for a total of 40 s from mesial, distal, incisal, and cervical directions as 10 s for each. After curing, the specimens were then placed in an incubator in water at 37°C for 24 h.

### 2.3. Shear Bond Strength Test

A universal testing machine (5848 MicroTester; Instron, Norwood, MA, USA) was used to evaluate SBS (at a crosshead speed of 1 mm/min). Shear bond strength was determined as registering shear force between bracket and bonding surface until debonding occurred. A digital caliper was used to determine the cross-sectional area of the brackets by measuring their width and length (and computing the area measured) [8]. The maximum force applied before debonding was divided by the cross-sectional area of the mandibular incisor ceramic bracket. Thus, the data was obtained as megapascals (MPa). Different investigators performed the SBS test and specimen preparation including the grouping of the specimens and all were blinded to the study protocol.

### 2.4. Determination of the Failure Mode

After SBS evaluation and debonding, both brackets and CAD/CAM block specimen surfaces were analyzed at 40x magnification (Zeiss Stemi SV11 Stereoscope; Carl Zeiss, Thornwood, NY, USA) to determine the mode of failure. Scanning electron microscope (SEM) (Quanta Feg 250, ThermoFisher Scientific, Hillsboro, Oregon, USA) images were also evaluated at ×200 magnification. Adhesive Remnant Indexes (ARI) were used to classify the failure mode described by Artun and Bergland [9]:

(0) No adhesive left on the VE and LU specimens
(1) Less than half of the adhesive remaining adhered to VE and LU specimens
(2) More than half of the adhesive left on the VE and LU specimens
(3) All adhesive remaining adhered to VE and LU surface

### 2.5. Statistical Analysis

To compare mean SBS among different groups of the study, statistical analysis was conducted by means of two-way analysis of variance (ANOVA). In the next step (post hoc), the Tukey test was used to compare the groups. Chi-square (*X*^2^) test was used to determine whether there were any significant differences in the ordinal ARI values. The statistical significance was set at the 0.05 probability level. All statistical analyses were performed using SPSS software (SPSS, Version 15.0; SPSS, Chicago, IL, USA).

## 3. Results

The mean and standard deviations of the shear bond strength values (MPa) are presented in Table 2. According to the two-way ANOVA results, the CAD/CAM block types and surface treatments had significant effects on the shear bond strength (*p* < 0.05). Also, there was a significant interaction between type of CAD/CAM block and surface treatment (*p* < 0.05).

**Figure 1:**
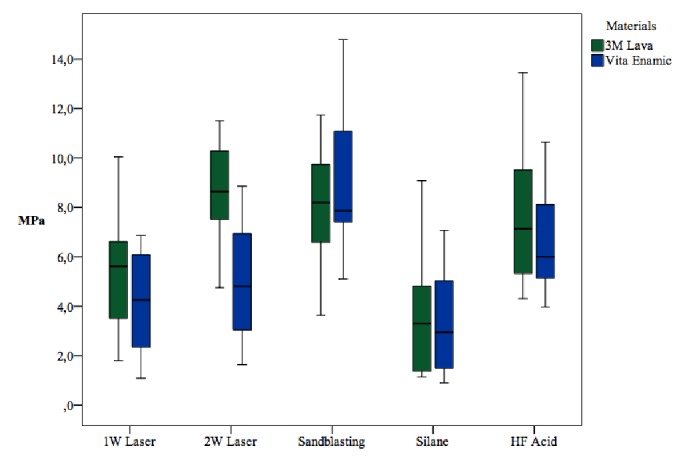
Shear bond strength (MPa) of ceramic brackets to CAD/CAM interpenetrating network composite and nanoceramic composite evaluated in the present study.

Figure 1 and Table 2 show shear bond strength (MPa) of ceramic brackets to CAD/CAM interpenetrating network composite and nanoceramic composite evaluated in the present study. The bond strength values ranged from 3.48 to 8.71 MPa for LU. The lowest bond strength values were found in the silane-treated group (*p* < 0.005). The 2 W laser application resulted in significantly higher bond strength than the 1 W laser application (*p* < 0.05). The bond strength values ranged from 3.35 to 8.92 MPa for VE. For VE specimens, sandblasting gave the highest bond strength (*p* < 0.05). As in the LU silane-treated group, VE samples treated with silane had the lowest bond strength values (*p* < 0.05). In general, sandblasting and HF acid surface treatment methods were more effective at achieving durable bond strength for the two types of CAD/CAM specimens.

**Figure 2:**
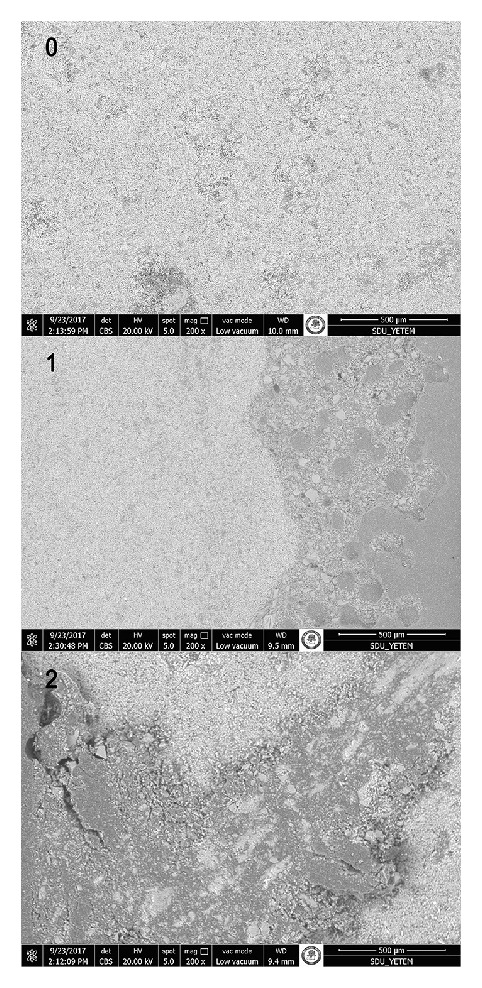
Scanning electron microscope images of examples to Adhesive Remnant Index scores in the present study: 0, no adhesive left on the CAD/CAM specimens (LU = 5, VE = 10); 1, less than half of the adhesive remaining adhered to CAD/CAM specimens (LU = 46, VE = 43); 2, more than half of the adhesive left on the CAD/CAM specimens (LU = 9, VE = 7).

Table 3 shows the ARI scores and Chi-square test results. The Chi-square test showed that there were significant differences in ARI between surface treatments of LU (*x*^2^ = 26.043) and VE (*x*^2^ = 30.110) (*p* < 0.05). The ARI scores revealed that less than half of the adhesive remaining adhered to VE and LU specimens (Score 1) for all surface treatment groups.  Figure 2 shows scanning electron microscope (SEM) images of examples to ARI scores.

## 4. Discussion

As with other dental treatments, adhesive interfaces between tooth/restoration surfaces and brackets have a great influence on the success of orthodontic treatment. Thus, different methods have been proposed to overcome the bonding problem of orthodontic brackets to dental restorative materials and different surface treatments have been suggested [3, 5, 10–17]. It was showed that abrading glazed porcelain mechanically or chemically may cause better bond strength [18] whereas other researchers have implied that deglazing of the porcelain surface has no advantage [5, 19]. Despite this, a ceramic restoration having a surface treatment for bracket bonding must be glazed after removal of the brackets. It has also been shown that the use of diamond burs on porcelain to produce a rough surface for bracket bonding may cause crack initiation and propagation [20, 21]. In this aspect, one of the advantages of new generation polymer-based CAD/CAM materials for an adult orthodontic patient may be the lack of need to remove restorations for a glazing procedure, unlike conventional dental ceramics. Besides this, the finishing and polishing procedures of IPNs and NCC are possible intraorally after the completion of orthodontic treatment. In this point of view, new generation esthetic dental materials may be an alternative to dental porcelain given their intraoral finishing and polishing procedures, similar characteristics to natural tooth structure and bonding performance to brackets. Thus, the purpose of the present study was to investigate whether the bonding performance of ceramic brackets to two different CAD/CAM restorative materials that have different surface treatments is sufficient for orthodontic treatment.

Reynolds [22] showed that the optimal bond strength between orthodontic bracket and porcelain is in the range 6–8 MPa. However, the suggestion that higher adhesive bond strength may lead to better orthodontic treatment outcome is debatable because excessive bonding strength may destroy and damage the restorative surfaces while debonding [23]. This means that adhesive bond strength between brackets to dental materials or tooth surfaces must be optimally high for successful orthodontic treatment but low enough not to cause damage or crack propagation to bonding surfaces during the debonding procedure. Thurmond* et al*. [24] found that bond strength between ceramic and adhesive bonding more than 13 MPa may cause fracture of the ceramic restoration. In the present study, the adhesive bond strength between polymer-based materials and ceramic brackets ranged from 3.35 to 8.92 MPa. The bond strength values determined in all groups of the present study were lower than the limit value (13 MPa) of ceramic fracture. According to the results of ARI scores, the most often failure (Score 1) is where less than half of the adhesive remaining adhered to VE and LU specimens. Thus, this means that there is no fracture on the restorative materials. However, the limit force for debonding of ceramic brackets without fracture of polymer-based CAD/CAM materials should be evaluated since there is a lack of knowledge about this subject in the dental literature.

In the past, different methods to overcome the bonding problems of restorative materials to brackets were suggested [7, 13]. Ajlouni* et al. *[18] showed that abrading glazed porcelain mechanically or chemically may cause better bond strength whereas other researchers have implied that deglazing of the porcelain surface has no advantage [5, 19]. The researchers [5, 19] also suggested silane bonding instead of chemical and mechanical abrasion. In a study that evaluated bonding performance of VE to brackets, Elsaka [7] found that silica coating enriches the adhesive bonding of both ceramic and metal brackets to VE. However, the bond strength value caused by silica coating (15.25 ± 3.31 MPa) was even greater than porcelain fracture risk value (13 MPa). In the same study [7], hydrofluoric acid group showed (11.87 ± 2.13 MPa) bond strength to ceramic brackets. Although the result of HF acid application for VE (11.87 ± 2.13) in Elsaka's study [7] is greater than the present study (8.92 ± 2.77), both studies' bond strength values imply that hydrofluoric acid application to VE specimens may cause a bond strength that is high enough for optimum bracket bonding and low enough to prevent the restorative material fracture.

According to the results of the study, bond strengths of LU and VE to the ceramic brackets were similar after the same surface treatments except 2 W laser application. The reason of lower bond strength in VE than LU after 2 W laser application may be originated from the effects of 2 W laser on the different microstructures of these two materials. LU is also defined as indirect composite CAD/CAM material. This means that the microstructure of LU was mainly composed of composite resin. However, VE was also defined as hybrid ceramic, composed of 86% ceramic reinforced by a 14% acrylate polymer network, with both networks penetrating completely. The lower bond strength of VE than LU after 2 W laser application may have resulted from VE being more indurate to laser application because of dominant ceramic content in the microstructure. In the present study, sandblast and HF acid applications generated better bond strength values than silane and 1 W laser applications. The result may be originated from the sandblasting, and HF revealed more suitable micro retentive surface for ceramic bracket bonding. HF acid treatment generates uniformly distributed pores and shallow irregularities originated from reaction between HF acid and silica components of the ceramic. Blasting with aluminum-oxide particles with high pressure also causes micro retentive surfaces. Thus, sandblasting and HF acid application may be promoted by better bond strength for both LU and VE in the present study in these ways.

In the LU groups, all surface treatments showed desired bracket bonding values ranging between 6 and 8 MPa, [22] except the silane treatment (3.48 ± 2.43 MPa) and 1 W Laser (5.29 ± 2.38). Sandblasting and hydrofluoric acid application resulted in sufficient shear bond strength for orthodontic treatment in the VE and LU groups. Silane application alone was not effective in either the VE or LU groups. According to the results of the present study, it may be suggested that sandblasting or hydrofluoric acid application may create enough ceramic bracket bonding for both VE and LU.

## 5. Conclusions

(1) According to the results of this study, surface treatment types affect the bond strength between ceramic brackets and CAD/CAM restorative materials investigated in the present study.

(2) Among the surface treatments evaluated, sandblasting and hydrofluoric acid application resulted in sufficient bond strength to ceramic brackets in both VE and LU.

## Figures and Tables

**Table 1:** Materials used in the present study.

Materials	Brand Names	Manufacturer	Lot
Interpenetrating network composite (IPN)	Vita Enamic (VE)	Vita Zahnfabrik, Cuxhaven, Germany	41470
Nano-ceramic composite (NCC)	Lava Ultimate (LU)	3M ESPE Dental Products, St. Paul, MN, USA	N613657
Mandibular incisor ceramic brackets	Damon Clear	Ormco Corp, Orange, CA, USA	051766799
Acrylic resin	Paladent	Heraeus Kulzer GmbH, Grüner Weg, Hanau, Germany	012427
Hydrofluoric acid	Ultradent Porcelain Etch	Ultradent Products, South Jordan, UT, USA	BCB97
Silane	Ultradent Silane	Ultradent Products, South Jordan, UT, USA	BDVRD
Orthodontic adhesive	Transbond XT 3M	Unitek, Monrovia, CA, USA	HV4ZV

**Table 2:** Means of shear bond strength (MPa) (±SD) between LU or VE blocks and ceramic brackets after different surface treatments.

	SHEAR BOND STRENGTH (MPa)				
	1 W Laser	2 W Laser	Sandblast	Silane	HF
Lava Ultimate (LU)	5.29 (±2.38)^ab^	8.71 (±2.03)^c^	8.07 (±2.52)^bc^	3.48 (±2.43)^a^	7.710 (±2.94)^bc^
Vita Enamic (VE)	4.19 (±1.98)^ab^	5.11 (±2.42)^ab^	8.92 (±2.77)^c^	3.35 (±2.09)^a^	6.67 (±2.11)^bc^

Means followed by different lowercase letters in each row for LU or VE differ statistically by Tukey's HSD test at 5%.

**Table 3:** Frequency distribution of Adhesive Remnant Index (ARI) scores and Chi-square test results.

Material	Surface	ARI Scores				*x* ^2^	Asymp.
	Treatments	0	1	2	3		Sig. (2-sided)
**Lava Ultimate (LU)**	1 W Laser	2	10	0	0		
	2 W Laser	0	12	0	0		
	Sandblast	0	6	6	0	26.043	0.001
	Silane	3	9	0	0		
	HF	0	9	3	0		

**Vita Enamic (VE)**	1 W Laser	1	11	0	0		
	2 W Laser	0	12	0	0		
	Sandblast	0	7	5	0	30.110	0.000
	Silane	6	6	0	0		
	HF	3	7	2	0		

## Data Availability

The data used to support the findings of this study are available from the corresponding author upon request.
